# The role and significance of graphene oxide in the remediation of micro- and nanoplastics from the environment

**DOI:** 10.1039/d5ra04896f

**Published:** 2025-10-03

**Authors:** Faiza Zainab, Ammara Aftab, Sadullah Mir, Nasser S. Awwad, Hala A. Ibrahium

**Affiliations:** a Department of Chemistry, COMSATS University Islamabad Park Road, Tarlai Kalan Chak Shahzad Islamabad Pakistan Sadullahmir@comsats.edu.pk +9203336781744; b Chemistry Department, King Khalid University P.O. Box 960, AlQura'a Abha Saudi Arabia; c Biology Department, King Khalid University P.O. Box 960, AlQura'a Abha Saudi Arabia

## Abstract

Micro- and nanoplastics (M/NPs) are widespread environmental pollutants arising from the increased use of plastics, presenting significant threats to human health and freshwater ecosystems. These particles are derived from both secondary and primary sources, including the breakdown of larger plastic debris and industrial abrasives, and cosmetics. After being released, M/NPs move through the air, water, and soil, where they persist, bioaccumulate, and interact with biological systems, potentially causing toxicity, inflammation, and oxidative stress. This study thoroughly addresses the origins, environmental routes, and health impacts of M/NPs, as well as the most current remediation strategies. Physical, chemical, biological, and hybrid therapeutic techniques are evaluated critically, with adsorption receiving special attention due to its efficiency and simplicity of usage. Graphene oxide (GO), a potential carbon-based adsorbent with a large surface area, several oxygen-containing functional groups, and a remarkable removal capability (up to 617.28 mg g^−1^ for polystyrene microplastics), receives special attention. Along with a comparison with other adsorbents, the review discusses GO's structural properties, synthesis procedures (including the Hummers' process), and adsorption mechanisms. This study contributes to the development of cutting-edge, environmentally friendly water treatment technologies by combining new research and emphasising the potential of GO-based materials for effective M/NP remediation in aquatic settings.

## Abstract 2

Ecotoxicological impact of M/NPs.

## Graphical abstract

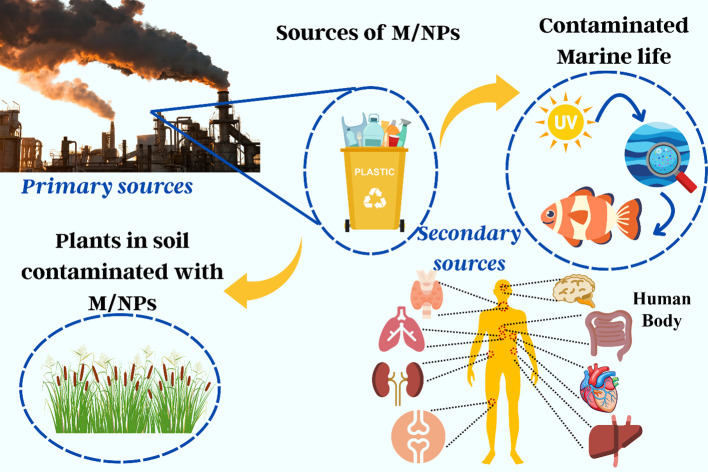

## 1. Introduction

Plastics are polymeric materials characterised by a high molecular mass composite. These hybrid materials are made by adding additives to a base polymer to enhance its properties.^1^ These materials are utilised in various industries, such as buildings, food packaging, medical equipment, transportation, and electrical insulation.^2^ This is because plastics are affordable, robust, lightweight, and adaptable. Global data indicate that plastic production reached 367 million tons in 2020, with the rate of output increasing steadily.^3^ Plastic is a synthetic substance that was created for human luxury, but its use is becoming increasingly problematic daily. Presently, the consumption of plastic has surged from 5 million tons in 1950 to 100 million tons today, marking a 20-fold increase. It was produced as a result of the 50% increase in single-use throwaway plastics.^4^ Although plastic materials have many benefits in daily life, their limited biodegradability, improper use, and ineffective disposal contribute to environmental degradation.^5–7^ Furthermore, many plastics are buoyant in fresh and marine waters and have low density analogously, which makes it easy for currents to carry them.^8–13^ Plastic garbage finds its way into aquatic environments where it is broken down physically, chemically, and biologically through various types of processes such as abrasion, UV light, hydrolysis, oxidation, and microbial breakdown. It is well accepted that exposure to sunshine, air, and water, which together generate an infinite amount of microplastics, triggers photooxidation and hydrolysis, which starts the breakdown process. Plastic objects may degrade into microplastics, which may subsequently degrade into nanoplastics under the influence of chemical, biological, physical, and environmental factors.^14–17^ Micro- and nanoplastics are generally classified into two main groups: primary and secondary.^18^ The major sources of microplastics include synthetic fabrics, industrial blast cleaning, and cosmetics wastewater.^19–21^ Both human activity and natural weathering produce secondary microplastics. Through atmospheric deposition, marine fisheries, and treatment plant effluent, microplastics can find their way into aquatic habitats.^22–25^ Because of their enlarged surface area and enhanced adsorption capacity, the smaller particles, such as micro and nanoplastics, exhibit a greater ability to interact with additional pollutants, encompassing pathogens and heavy metals. Based on their chemical makeup, microplastics persist in the aquatic environment for an extended period after they enter aqueous environments.^26–30^ Microplastics can manifest in various forms such as pellets, fibres, fragments, film foams, *etc.* Moreover, they can either float on the water's surface or attach to plants, rocks, and sediments. Microplastics can also range in size up to 5 mm. They can reach organisms through many entrance points and move up the food chain, which can harm aquatic life in many ways, including through growth, development, reproduction, and survival.^31–35^ Among all plastic trash materials, micro- and nanoplastics pose the greatest threat and need to be addressed.^36–38^ Human health and the environment are under serious threats due to single-use PPE (personal protective equipment) made of nonbiodegradable materials, which arises from the generation of micro- and nanoplastics.^39–42^ One current area of study is the removal of microplastics from aquatic habitats. For their removal from the environment, various degradation methods have been employed.^43–45^ Researchers have discovered the use of ultrahigh-temperature composting, microbial decomposition, and photocatalytic degradation as ways to eliminate microplastics from water bodies. Microbial degradation occurs at a moderate rate, whereas photocatalytic degradation necessitates a prolonged irradiation period.^46,47^ Composting at extremely high temperatures works well, but there are still safety issues. Aquatic ecosystems are colonised by microplastics, which create persistent biofilms that improve the adsorption and break down organic contaminants.^48^

Removing micro- and nanoplastics is difficult; wastewater treatment removes only 98.41% of plastics. Nonetheless, 83% of drinking water samples are contaminated by 65 million microplastic particles that persist every day. Effective techniques for removing microplastics, including membrane devices and filtration, are being studied by researchers. Most microplastics are eliminated by tertiary treatment, although secondary treatment is problematic. There is an immediate need for high-efficiency and reasonably priced techniques to remove microplastics.^49,50^ One popular technique for treating microplastics in water is adsorption. Adsorption is a common technique for eliminating tiny molecules from water and is inexpensive, accessible, and highly effective in terms of purification. New materials such as graphene, TiO_2_ (titanium dioxide), and CNTs (carbon nanotubes) have been produced to address the issue of wastewater treatment.^51,52^ Graphene has been utilised extensively as an adsorbent due to its great modifiability, abundant oxygen functional groups, and enormous surface area.^53,54^ Xu *et al.* employed graphene for the disinfection of bisphenol from water and achieved an efficient adsorption capacity of 182 mg g^−1^.^55,56^ However, two-dimensional (2D) graphene tends to agglomerate in an aqueous solution due to the robust π–π stacking contact between sheets. When built into a three-dimensional (3D) structure, graphene offers help for separating the mixture of solid and liquid after adsorption, avoiding agglomeration, and enhancing the diffusion and adsorption of contaminants.^57–60^ To eliminate microplastics, a common kind of plastic pollution, researchers are employing 3D GO as an adsorbent. Nowadays, 3D RGO adsorbents exhibiting a remarkable maximum adsorption capacity of 617.28 mg g^−1^ have gained significance for the elimination of polystyrene (PS) microplastics. As far as the researchers are aware, no relevant review papers have yet been published for the remediation of micro- and nanoplastics from aqueous environments by employing a graphene oxide-based adsorbent. To provide context, the following section offers a brief overview of the sources, transport pathways, and environmental and health impacts of micro- and nanoplastics.

### 1.1 Overview of micro- and nanoplastic pollution

**Fig. 1:**
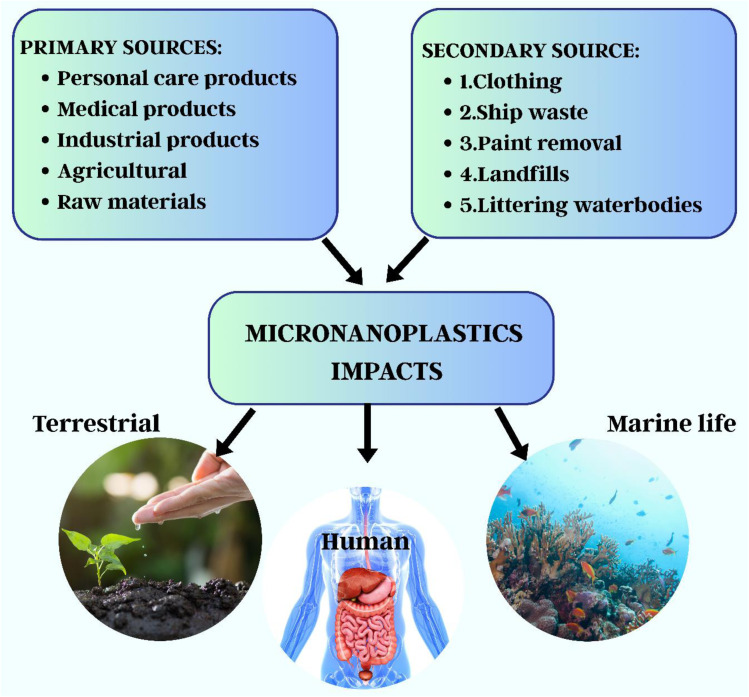
Primary and secondary sources of micronanoplastics.

**Fig. 2:**
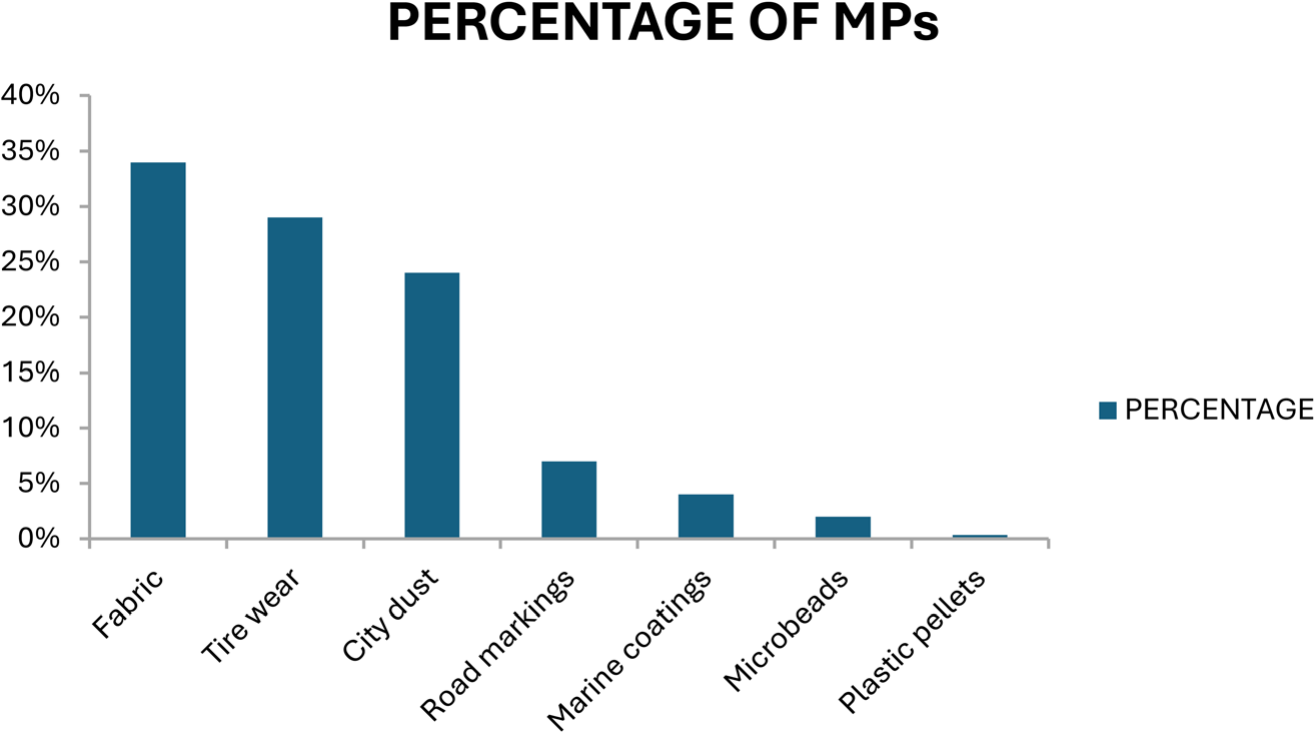
Global sources of microplastics.

Microplastics (MPs), or plastic particles smaller than 5 mm in size, were detected in phytoplankton samples, including microfibers and microbeads, in 1960. They exhibit a wide range of surface characteristics, colours, polymer kinds, and morphologies.^61–67^ MPs are derived from primary sources, which are purposely manufactured particles such as plastic nurdles, industrial abrasives, and microbeads in cosmetics, and secondary sources, which are formed when larger polymers degrade due to oxidation, UV radiation, and mechanical forces.^68–70^ While secondary MPs account for 70–80% of all microplastics worldwide, primary MPs are regularly generated as a result of tire wear, washing, and industrial discharge.^5,6,71^ Nanoplastics (NPs), which are typically less than 1000 nm in size, can adsorb hazardous chemicals and penetrate biological systems more deeply than MPs.^72–76^ Their ingestion can disrupt aquatic creatures' growth, development, and hormonal balance, raising the ecological danger.^77–79^ M/NPs reach the environment through primary sources (such as abrasive and cosmetic leaching) and secondary microplastic degradation, as illustrated in Fig. 1.^80–85^ These particles commonly accumulate in aquatic settings after flowing through wastewater systems.^86–88^Fig. 2 shows that the primary sources of MP pollution are synthetic textiles (34%), tire wear (29%), urban dust (24%), road markings (7%), marine coatings (4%), microbeads (2%), and plastic pellets (0.3%).^70,86,89–93^ However, because of the overlapping properties of primary and secondary particles, identifying and quantifying sources is challenging, stressing the need for more research into their origins, behaviour, and environmental destiny.^94–96^

### 1.2 Pathways and persistence of M/NPs across ecosystems

**Fig. 3:**
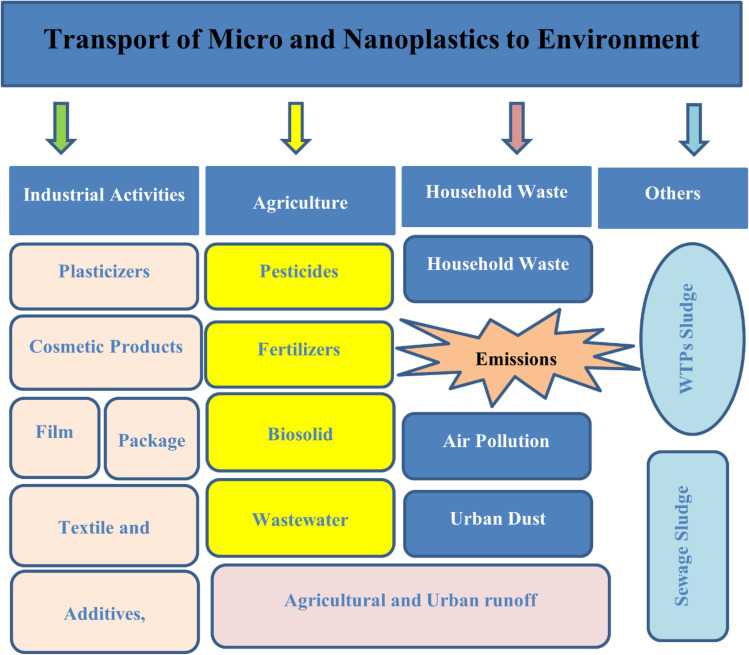
Transport of M/NPs in the environment.

M/NPs originate from several sources, including synthetic fabrics, urban runoff, tire wear, and wastewater treatment facilities (WWTPs), which both filter and accidently release M/NPs into the environment.^97–102^ These particles move through soil, freshwater systems, and the atmosphere, and their distribution in terrestrial and marine ecosystems is affected by deposition and resuspension processes.^65,67,103^ Because polymers disintegrate when exposed to sunshine and oxygen, synthetic fibres and polystyrene (PS) particles are prevalent in maritime habitats.^104–109^ While sewage sludge and agricultural runoff pollute the land, WWTP biosolids and effluents are significant entry routes into aquatic systems. Their endurance in soil and food systems is proven by detection techniques such as Coulter counters and nanoparticle tracking, particularly for PS, the fourth most prevalent MP.^110–112^ Their persistence in soil and food chains is demonstrated by detection techniques such as Coulter counters and nanoparticle tracking, particularly for PS, the fourth most common MP in agricultural soils Fig. 3.^113–120^ Successful mitigation methods need an understanding of their environmental transport.

### 1.3 Ecotoxicological and health impacts of micro- and nanoplastics

**Fig. 4:**
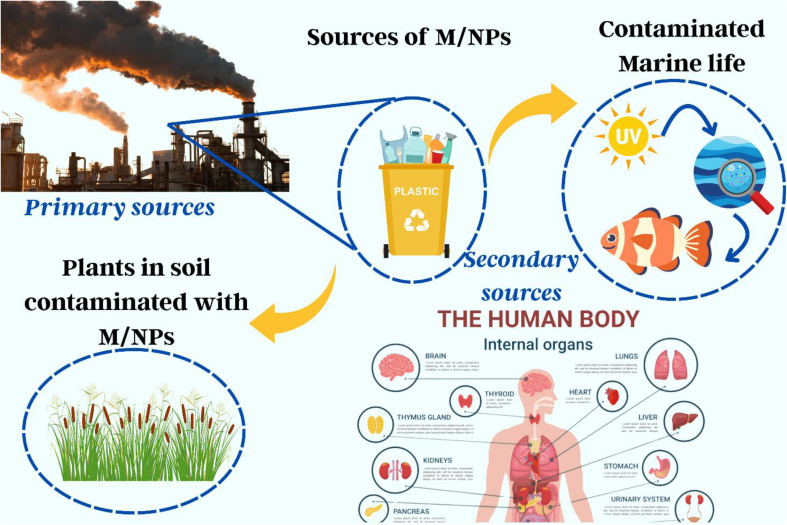
Ecotoxicological impact of M/NPs.

**Fig. 5:**
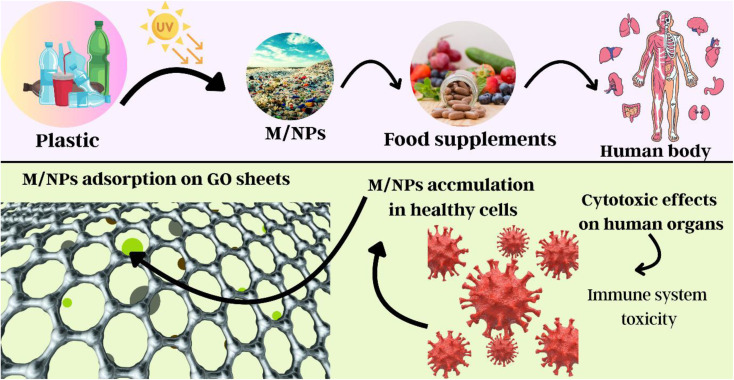
Pathways and health impacts of micro- and nanoplastics in humans, from environmental exposure to biological effects and associated disorders.

The consequences of micro- and nanoplastics (M/NPs) on aquatic life, terrestrial ecosystems, and human health are wide and diverse. M/NPs concentrations in terrestrial contexts are often 4–20 times greater than in aquatic systems, affecting soil bulk density, water retention, and plant physiology, and therefore altering crop development, nutrient dynamics, and soil structure in Fig. 4.^6,121–140^ Numerous critical investigations have examined the concept that microplastics significantly transmit hydrophobic organic compounds (HOCs) to marine creatures. In contrast to natural exposure pathways such as prey, Koelmans *et al.* (2016) found that HOC absorption from microplastics is negligible. Desorption research and bioaccumulation models have led to a consensus that microplastics have a negligible influence on HOC transmission in maritime settings.^141^ M/NPs accumulate in sediments and biota in marine settings due to oxidative stress and enzyme inhibition, compromising feeding, immunity, and neurological functions in animals such as nematodes and zebrafish.^142–152^ Furthermore, through trophic transfer and contaminated food, these plastics can bioaccumulate from phytoplankton to people, potentially causing organ toxicity and DNA damage.^125,153–163^ Humans are mostly exposed through the consumption of contaminated food and water (such as salt, honey, and vegetables), which can accumulate in organs such as the liver, brain, and lungs, causing long-term inflammatory and cellular damage as illustrated in Fig. 5.^164–171^ These findings highlight the critical importance of comprehensive M/NP pollution monitoring and mitigation in all environmental compartments.

## 2. Comparative study and publication analysis

**Fig. 6:**
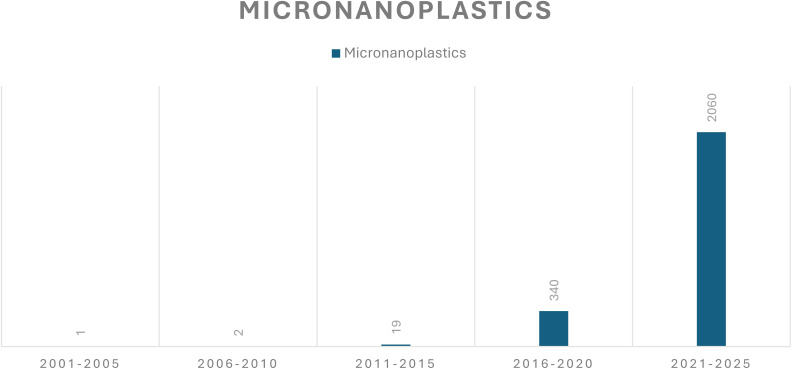
Publication analysis of research articles dated 18 Aug 2025.

Ali Imran, *et al.* provided a comprehensive analysis of filtration technology for the removal of microplastics, particularly emphasising the filter media characteristics and other environmental factors on removal efficacy.^172^ Wang Xiaojie, *et al.* investigated the degradation of micro- and nanoplastics by focusing on advanced oxidation processes like photolysis, photocatalysis, and ozone oxidation.^173^ Sutrisna Putu Doddy, *et al.* discussed the membrane technology, particularly addressing membrane bioreactors for efficient removal of nanoplastics.^174^ Yu Tingting, *et al.* highlighted the use of nanomaterials as adsorbents, catalysts, and membranes in the removal of micro- and nanoplastic and their potential hazards and limitations in the research.^175^ In contrast to the above studies represented here, our review focuses on the application of 2D nanomaterials, specifically graphene oxide (GO), as an adsorbent for the removal of micro- and nanoplastics (MNPs). By representing the superior adsorption capacity of GO for binding with microplastics for efficient removal. We highlighted a novel, more efficient approach for micro nanoplastic degradation studies. The publication analysis chart of research articles is shown in Fig. 6.

## 3. Remediation of micro- and nanoplastics by distinct methods

**Fig. 7:**
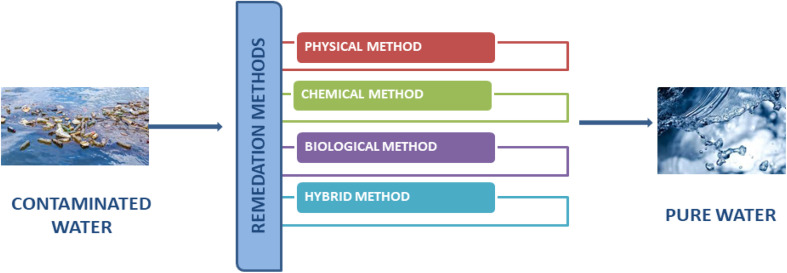
Various remediation technologies for treatment of micro- and nanoplastics contaminated water.

In this critical analysis, the economic and environmental viability of many physical, chemical, and biological remediation techniques, including cutting-edge hybrid technologies, primarily microbial fuel cells and electrolysis cells for the elimination of M/NPs from aqueous solution, is covered.^176–178^ It also looks into how sustainable these technologies will be in the long run.^179^ Based on performance efficiency, techno-economic analysis (TEA), and life cycle assessment (LCA), various technologies are compared (Fig. 7).

**Fig. 8:**
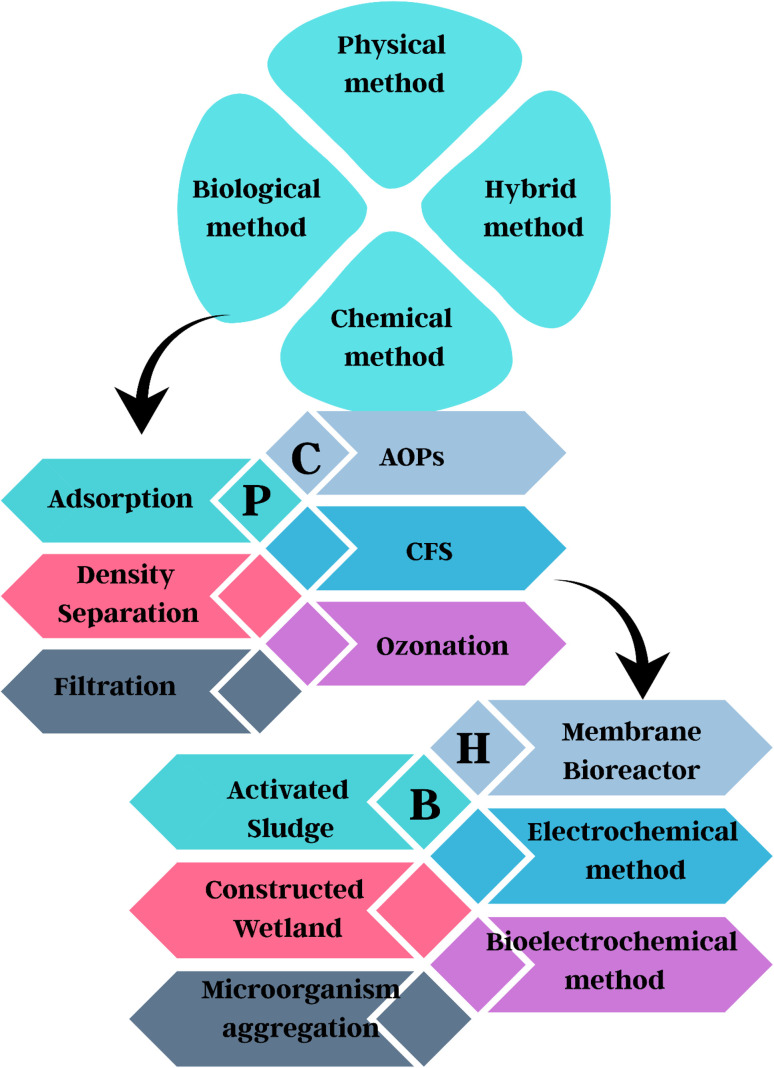
Flowsheet of physical, chemical, biological, and hybrid methods used for the remediation of micro- and nanoplastics.

In addition, the removal procedures of different technologies have been explained. This information focuses on which technology mineralises, degrades, or phases out MNPs completely, offering insight into the process of choosing the best technology for M/NP elimination.^180^ Additionally, the advantages, disadvantages, and difficulties of the present and prospects for various MP removal methods have been discussed. Fig. 8 shows the various physical, chemical, biological, and hybrid treatment methods employed for the removal of M/NPs.^63,181^

### 3.1 Physical treatment methods: adsorption, density separation, and filtration

Adsorption is a well-studied physical approach for M/NP elimination due to its simplicity and efficacy. Granular and powdered adsorbents, such as Zn–Al layered double hydroxides (LDH), have been found to remove MPs from deionised water by up to 96%.^182–184^ Despite repeated application, chitin–graphene oxide (ChGO) sponges have achieved 90% clearance. Furthermore, biobased adsorbents such as aerogels and magnetic biochar are gaining popularity.^182–184^ Nii Ashitey Anuwa-Amarh, *et al.* (2024) examined carbon-based adsorbents such as graphene, activated carbon, biochar, and carbon nanotubes for the removal of microplastics in wastewater. They discussed how interactions such as π–π and electrostatic forces promote adsorption, emphasising the relevance of surface area, porosity, and chemical characteristics. In laboratory testing, improved adsorbents typically achieved removal rates over 90%.^185^ Adsorbents usually lose their efficiency in complicated water matrices, and the disposal of saturated materials can cause secondary contamination. Furthermore, regeneration and reuse are difficult to do on an industrial scale.^180^

Density separation is based on particle density changes. M/NPs generally have densities of 0.8–1.4 g cm^−3^. Lighter polymers can float and be separated by adding salts such as NaCl and NaI, which create density gradients.^179,186–188^ Crutchett *et al.* used ZnCl_2_ solutions to separate dense microplastics such as PA, PVC, and PET from sediments, recovering almost 95% of the total. The approach had an average recovery rate of 96%, indicating good efficiency and repeatability.^189^ When the densities of microplastics match those of sediments or solution, density separation is restricted, resulting in partial recovery to the enormous volumes of salt required; the technique is not economically viable for high-density polymers such as PET or PVC.^190–192^ Common salt solutions can also be costly, hazardous, and ineffective, and the technique is often laborious and time-consuming.^193,194^

M/NPs are frequently removed from environmental water and liquid food matrices using disc, sand, and membrane filtration systems. Sand filtration alone can remove up to 73% of the material; when pre-coagulation is used, this number rises to about 90%. Depending on the system configuration and particle properties, membrane-based filtration systems, such as ultrafiltration and nanofiltration, have shown removal efficiencies of up to 96.77% for MPs and approximately 90% for NPs.^195–204^ According to Mishra Sunanda *et al.*, membrane bioreactor filtration offers great promise for reducing microplastics in treatment systems. It removed microplastics from wastewater with 96% efficiency, mostly by size exclusion and mechanical straining.^205^ Despite their great efficiency, filtration processes provide significant operating hurdles. Membrane fouling is an ongoing issue that increases maintenance frequency and energy usage. Physical and chemical stresses can also damage filters, particularly in long-distance water transport systems. Backwashing may return microplastics if not properly handled, whereas coagulation can improve removal but adds cost and complexity.^206,207^

### 3.2 Chemical treatment methods: advanced oxidation reactions, coagulation, flocculation, sedimentation, and ozonation

Advanced oxidation processes (AOPs) generate reactive oxygen species (ROS) like ˙OH and SO_4_˙^−^ that degrade MPs. Photocatalysis using TiO_2_ and ZnO has proven effective, while persulfate and Fenton oxidations have shown varying degrees of success.^195,200,208–216^ Kaswan Vipin *et al.* reviewed advanced oxidation strategies for wastewater treatment, focusing on photocatalytic ozonation, the Fenton process, and TiO_2_-based photocatalysis. They discussed the advantages and disadvantages of each strategy, as well as the processes, catalysts, and influencing variables.^217^ AOPs typically fail to fully decompose a wide range of organic pollutants. Combining AOPs improves hydroxyl radical production and therapeutic efficacy. Nonetheless, the total oxidation performance may be influenced by the chemistry of the water, including competing ions.^218^

**Fig. 9:**
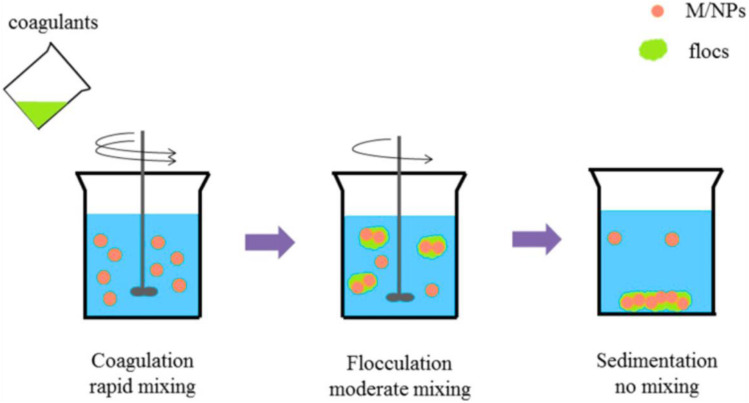
The coagulation, flocculation and sedimentation method to remove M/NPs. This figure has been reproduced from ref. 195 with permission from Elsevier, copyright 2025.

Coagulation, flocculation, and sedimentation (CFS) uses chemical flocculants to destabilise and aggregate M/NPs as shown in Fig. 9. Coagulants based on aluminium have proven to be very effective.^219–227^ Hofman-Caris *et al.* investigated the removal of metallic nanoparticles and nanoplastics with standard treatment procedures such as CFS, RSF, and GAC. The results showed that particle size, surface charge, water matrix, and cation presence all influence removal effectiveness, with GAC performing better for smaller particles and CFS favouring bigger ones. Negatively charged NOM impeded removal, whereas Ca^2+^ and Mg^2+^ facilitated it.^228^ The CFS process can effectively remove nano-CuO, but its efficiency is limited by the high coagulant demand and inconsistent performance of single metal coagulants. Not all M/NPs react equally to flocculants, and this process produces a lot of sludge. Additionally, synthetic chemicals may be toxic.^220,229–231^ While mixing organic and inorganic agents enhances removal, but adds complexity and expense. Floc formation is pH and stirring speed-dependent.^232^

Ozonation is an ozone-based oxidation procedure that reduces MPs to smaller fragments. The removal efficiencies vary from 53.8% to 89.9%.^179,215,233–236^ According to Wang Jie *et al.*, flocculation following ozonation pretreatment significantly enhanced microplastic removal from 40% to 91%. Ozonation altered the properties of microplastics by increasing the number of hydroxyl and carbonyl groups on their surfaces, boosting flocculation efficiency. Nonetheless, unoxidized floating microplastics persisted, demonstrating the importance of surface hydroxylation.^237^ Ozonation's usage for microplastic cleanup is limited due to its high operating costs and energy consumption. It may create hazardous byproducts like ketones and aldehydes. Its appeal is lessened by high running costs and the capacity to produce smaller, more mobile NPs. Because of its volatility, ozone must be generated locally. It may also reduce microplastics into smaller, possibly dangerous particles that are more bioavailable.^233^

### 3.3 Biological treatment methods: activated sludge method, microorganism aggregation, constructed wetlands

The activated sludge process (ASP) treats M/NPs as organic materials and adsorbs them with aerobic microbial populations. However, the technique does not mineralise plastics.^238–242^ Odunola *et al.* (2024) investigated the removal of microplastics in CAS and AGS systems, discovering that more EPS contact resulted in better efficiency at lower OLRs (96% in CAS and 94% in AGS). Confocal imaging indicated adsorption at the floc and granule surfaces, indicating that bigger microplastics were removed more effectively.^243^ Microplastics can be trapped in sludge using activated sludge processing (ASP), posing disposal and environmental risks. These microplastics enhance toxicity and reduce microbial diversity. Biological degradation in ASP remains mostly unsuccessful because of plastic recalcitrance and short retention durations. MPs accumulate in sludge, which is toxic and can remain in the environment for a long period.^244^

**Fig. 10:**
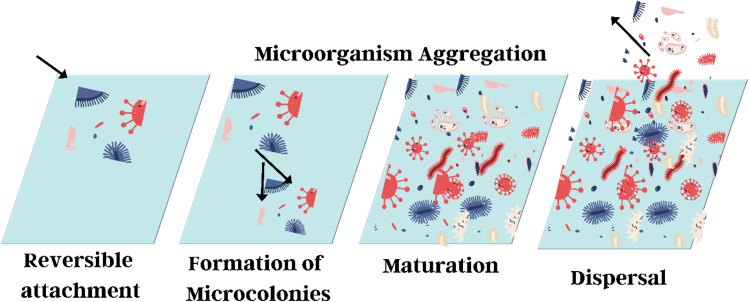
Illustration of microplastic aggregation facilitated by microorganisms through biofilm formation.

Microorganisms promote aggregate formation by binding to M/NPs. EPS from cyanobacteria and *Pseudomonas aeruginosa* has been shown in Fig. 10 to be effective at removing MP.^245–250^ Liu *et al.* utilised *Pseudomonas aeruginosa* for effective aggregation of MPs within its matrix, having a sticky nature, EPS, that is reversible and strongly associated with copper bioaccumulation on MPs.^251^ Romero *et al.* (2024) investigated interactions between polystyrene microplastics and *Pseudomonas aeruginosa*, showing that Psl exopolysaccharide promotes adhesion but not colloidal stability. MP aggregation was influenced by bacterial motility and water flow, preventing early sedimentation. Surprisingly, MP–PA aggregates remained motile, enhancing transport compared to passive diffusion.^252^ Environmental variability and biofilm growth conditions can limit the scalability and consistency of results. Since aggregation depends on c-di-GMP signalling, external stressors disrupting this pathway may reduce effectiveness. Environmental factors influence both microbial activity and EPS production. Not all polymers bond nicely.^251^

**Fig. 11:**
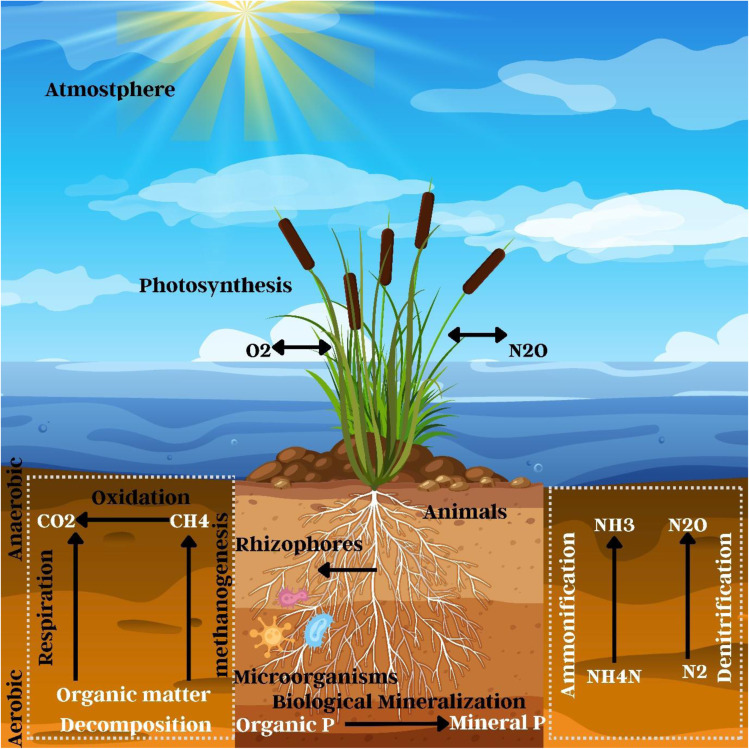
Remediation of micro- and nanoplastics by constructed Wetlands.

Constructed wetlands (CWs) use physical straining and biofilm formation to get rid of M/NPs, as shown in Fig. 11. Horizontal systems can remove up to 100% of MPs, whereas vertical flow CWs can remove up to 96%.^253–258^ Compared to surface flow systems, Chen *et al.* (2023) demonstrated that horizontal subsurface flow constructed wetlands (HSF-CWs) effectively cleaned up to 100% of microplastics. Microplastic size, shape, and substrate qualities all had an impact on retention, with biofilm filtering and adhesion playing a significant role.^256^ MPs have little influence on carbon removal, but they interfere with microbial activity and plant intake, disrupting the nitrogen cycle. There is a detrimental influence on phosphorus elimination, indicating that CW–MP interactions warrant additional exploration to alter the phase of plastics rather than breaking them down. Other issues include bioaccumulation in wetland species and land requirements.^17^

### 3.4 Hybrid treatment methods

By maximising the benefits and reducing the drawbacks of any individual treatment approach, a combination of multiple approaches can be used to remove M/NPs, as discussed in Table 1. The use of a membrane bioreactor, an electrochemical technique, and a bioelectrochemical method are the three most common approaches.

**Table 1:** Comparison of hybrid methods

Membrane bioreactor	Electrochemical method	Bioelectrochemical method
Micro–nanoplastics are efficiently removed from wastewater by membrane bioreactors; however, because of their high concentration of MNPs, additional treatment is required, which raises operating costs and presents problems with membrane fouling^259–263^	By producing hydroxyl radicals, electrochemical treatment methods such as electro-Fenton and electrochemical oxidation improve MP removal efficiency. On the other hand, electrocoagulation yields metal cations and achieves 58 ± 21% mineralisation of PS particles^77,264–267^	Acetaminophen and 4-aminophenol are two new micropollutants that bioelectrochemical systems (BESs) can remove. By comparing treatment technologies, best practices for field-scale removal of these pollutants can be determined^268–271^

In 2024, Corpuz *et al.* evaluated a novel electrochemically enhanced living membrane bioreactor (e-LMBR) for removing polyethene microplastics from wastewater. Despite the presence of MP, the e-LMBR retained high COD, NH_4_–N, and PO_4_–P removal rates while reducing MP by up to 95%. Electrochemical improvement outperformed typical MBRs made of less costly materials by stabilising effluent quality and minimising fouling.^272^ Its long-term viability and cost-effectiveness are jeopardised by excessive energy consumption and frequent electrode repair. Furthermore, the study did not conduct a full evaluation of performance in complex actual wastewater matrices.^264,273^

A complete assessment of removal efficiency, cost, sustainability, and environmental safety reveals that no single approach is uniformly superior. Although chemical and physical approaches are effective, they frequently consume a large amount of energy and generate secondary waste. Biological and hybrid systems, while requiring more research and field validation, provide long-term solutions. Techno-economic analysis (TEA) and life-cycle assessment (LCA) remain critical methodologies for determining the optimal technology for micro/nanoplastic cleanup.

## 4. Remediation of micro- and nanoplastics by adsorption

Researchers are currently looking for practical and cost-efficient ways to remove micro/nanoplastics (M/NPs) from water.^271^ While classic flocculation and ultrafiltration are less effective in removing polyethene microplastics, membrane-based approaches and algal bloom management measures show some promise.^274–276^ Although secondary and tertiary treatment methods have shown some success in studies conducted in Sweden and California.^277,278^ Adsorption has emerged as a popular technique due to its ease of use, affordability, and energy efficiency.^279^ This approach is especially effective at removing small plastic particles from wastewater. Novel materials such as graphene oxide (GO), titanium dioxide, carbon nanotubes (CNTs), and biochars are increasingly being explored for improved adsorption performance.

Table 2 compares the various microplastic-removal adsorbents based on their removal efficiencies, adsorption capacities, and processes. Graphene oxide (GO) has the highest adsorption capacity for polystyrene (PS) microplastics (617.28 mg g^−1^), attributed to electrostatic and π–π interactions.^280,281^ Other materials offer significant potential as well. Iron-modified biochar (FB) had an adsorption capacity of 206 mg g^−1^ and effectively removed 99.5% of PS *via* hydrophobic, π–π conjugation, and electrostatic interactions.^282^ CuNi@C nanocomposites achieved a removal efficiency of 99.18%, or a PS capacity of 38 mg g^−1^, using physical adsorption and electrostatic attraction.^283^ At low concentrations, ZIF-67, a metal–organic framework, has a 92.1% removal rate for PS microplastics.^284^ Polydopamine-enhanced magnetic chitosan composites had excellent removal efficiencies of 97.3% (PET), 94.6% (PE), and 92.3% (PS).^283^ Magnetic carbon nanotubes (CNTs) performed similarly well at higher doses, removing 85.8% of mixed microplastics such as PE, PET, and PA.^285^ Granular activated carbon (GAC) had a lower adsorption capacity of 2.20 mg g^−1^ for PS,^286^ whereas coffee grounds, despite being inexpensive and environmentally friendly, achieved only 74% removal or 4 mg g^−1^ adsorption capacity for PS *via* electrostatic interactions and hydrogen bonding.^290^

**Table 2:** Comparison of different adsorbents for MNPs removal

Adsorbents	M/NPs type	M/NPs concentration	Mechanism	Adsorption efficiency/amount	References
RGO	PS	600 mg L^−1^	Electrostatic and π–π interaction	617.28 mg g^−1^	53
ZIF-8@aerogel	PVDF, PS	0.5 g L^−1^	Electrostatic and hydrophobic interactions, H-bonding and van-der-Waals forces	91.4%, 85.8%	287
Chitin–GO sponge	PS	1 mg L^−1^		72.4–89.8%	288
Fe_3_O_4_ nanoaggregates	PS	4 mg mL	H-bonding	100% or 7.9 mg	289
Fe–kaolin	PVC, PS, PET	0.01 g mL^−1^	Electrostatic interaction	13.68 mg g^−1^	290
Cellulose fibres	PVAc	2 mg mL^−1^	Electrostatic interaction	99%	291
Zn–Al LDH	PS	250 mg L^−1^	Electrostatic interaction	100% or 164.49 mg g^−1^	182
Cellulose/Mg–Al LDH	PS	5 mg L^−1^	Hydrogen bonding, electrostatic interactions	6.08 mg g^−1^	292
Fe-modified FA	PS	30 mg L^−1^	Electrostatic attraction, complexation, π–π interactions	89.9 mg g^−1^	286
ZIF-67	PS	5 mg L^−1^		92.1%	61
Granular activated carbon (GAC)	PS	40 mg L^−1^	Electrostatic interactions	2.20 mg g^−1^	284
CuNi@C	PS	10 mg L^−1^	Physical adsorption, electrostatic attraction	99.18% or 38 mg g^−1^	283
Magnetic CNTs	PE, PET, PA	5 g L^−1^		85.8%	285
Iron-modified biochar (FB)	PS	10 mg L^−1^	Electrostatic interaction, π–π conjugation, hydrophobic interactions	99.5% or 206 mg g^−1^	282
Coffee grounds	PS	100 mg L^−1^	Electrostatic interactions, hydrogen bonding	74% or 4 mg g^−1^	293
Polydopamine-enhanced magnetic chitosan (PDA-MCS)	PET, PE, PS	300 mg L^−1^		97.3, 94.6, 92.3	285

Many of these materials have disadvantages, such as poor adsorption capabilities, the need for greater doses, or restricted reusability, despite the fact that some of them have good removal effectiveness. In contrast, GO is a very attractive option for practical water treatment applications due to its multifunctional surface chemistry, large surface area, strong affinity for various micro/nanoplastics, and remarkable adsorption efficiency.

### 4.1 Graphene oxide as an adsorbent

**Fig. 12:**
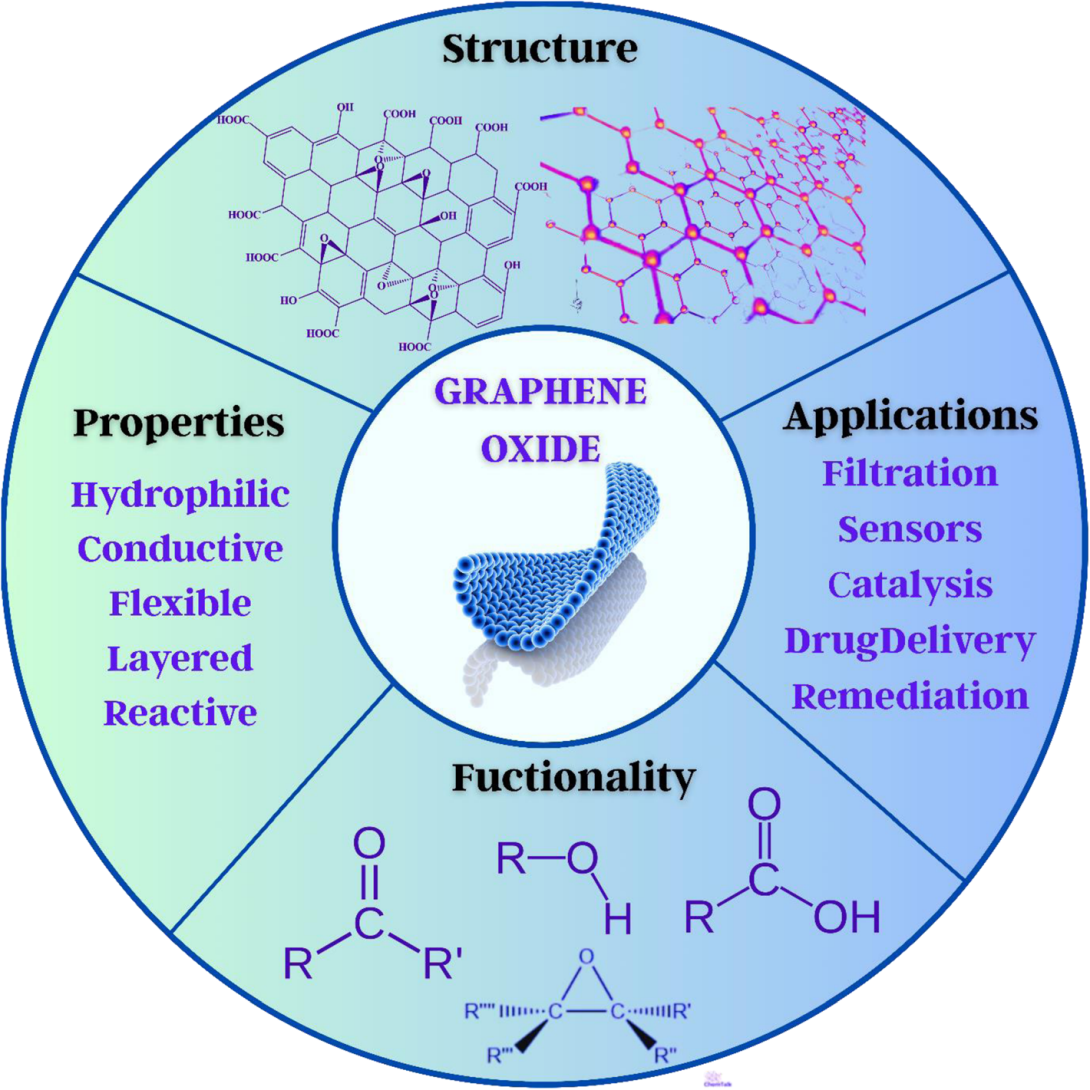
Structure, properties, and functional roles of graphene oxide.

The novel adsorbent material graphene has drawn interest because of its strong modifiability, abundant oxygen functional groups, and enormous specific surface areas, as shown in Fig. 12.^294–296^ It is a popular alternative for treating water contaminants because of its inexpensive cost and easy preparation method.^297^ According to previous studies, at pH = 6.5, magnetically reduced graphene oxide (RGO) has a far better adsorption capacity than activated carbon. Moreover, graphene oxide adsorbs Cu^2+^ in water, which is ten times greater than that of activated carbon. According to research, graphene has the greatest potential for carbon adsorption on BPA.^203,280,298,299^

### 4.2 Structure of graphene oxide

**Fig. 13:**
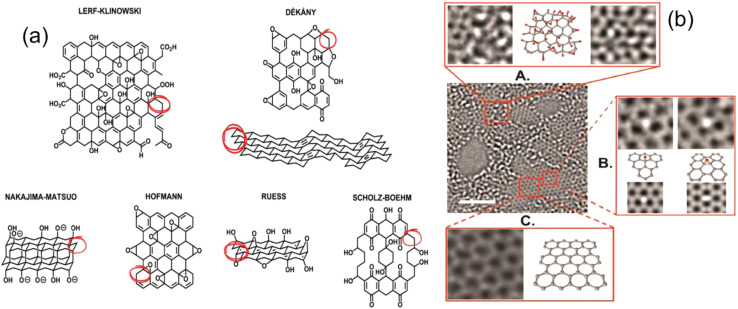
(a) Summary of structural models of GO (top: Lerf–Klinowski and Dékány models) (bottom: early structures examples, Nakajima-Matsuo model, Hofmann, Ruess, and Scholz–Boehm models) (b) high-resolution TEM image of suspended GO single sheet (A) a 1 nm^2^ extended region showing oxidized area of material (B) atomic structure for hydroxyl and (1,2) epoxy functionalities (C) 1 nm^2^ graphitic region from the exit plane. This figure has been reproduced from the ref. 309, 311 and 313, with permission from Elsevier, copyright 2025.

Early structural models of graphene oxide (GO) often overlooked carbon radicals and hydrogen bonding, resulting in CH and CH_2_ groups without hydrogen atoms. This omission is critical for understanding GO's reactivity.^300–302^ The widely used Lerf–Klinowski (LK) model, proposed in the late 1990s, gave a more realistic depiction of GO sheets by introducing two distinct domains: aliphatic (oxidized six-membered rings) and aromatic (non-oxidized benzene rings) was projected to describe the assembly of graphite oxide (GO) as shown by Fig. 13(a).^303^ While carboxyl and carbonyl groups are normally located on sheet edges, GO has a flat carbon structure with double bonds, aromatic regions, epoxide, and hydroxyl groups scattered across the basal plane.^304,305^ Advanced solid-state NMR investigations and ^13^C-labelled GO revealed fresh information on bonding topologies and 2D connectivity, while a Claisen-type rearrangement revealed the existence of allylic alcohols on GO surfaces.^306^ Tamás Szabó *et al.* extended the LK model by proposing a modified Scholz–Boehm structure for highly oxidised GO, complete with oxo groups and more diverse functional areas.^307,308^ Tamás Szabó concept works better for most kinds of GO and is more universal.^307^ This improved model considers the effect of synthesis factors, including temperature, pH, and hydration, on GO structure. Furthermore, Cai and Gao revealed the spatial segregation of carbonyl and carboxyl groups, revealing distinct reactivity areas within GO. However, Patrick P. Brisebois *et al.* reported that the Diels–Alder reaction has been successfully extended with graphene oxide. This reaction provides fundamental information for understanding the exact structure and chemical nature of graphene oxide.^309,310^ High-resolution TEM identified nanopores (<5 nm^2^) as shown in Fig. 13(b) generated during oxidation and exfoliation, as well as the release of CO and CO_2_ gases. XPS spectra^311,312^ suggest that these holes are surrounded by carbonyl groups.^311,312^ These structural features have a direct impact on GO's adsorption properties: functional groups such as carboxyl, hydroxyl, and epoxide groups are required for surface contact with M/NPs *via* electrostatic, van der Waals, and hydrogen bonding interactions. Understanding these structure–function correlations enables tailored GO production with greater selectivity for distinct plastic types.

### 4.3 Properties of graphene oxide

**Fig. 14:**
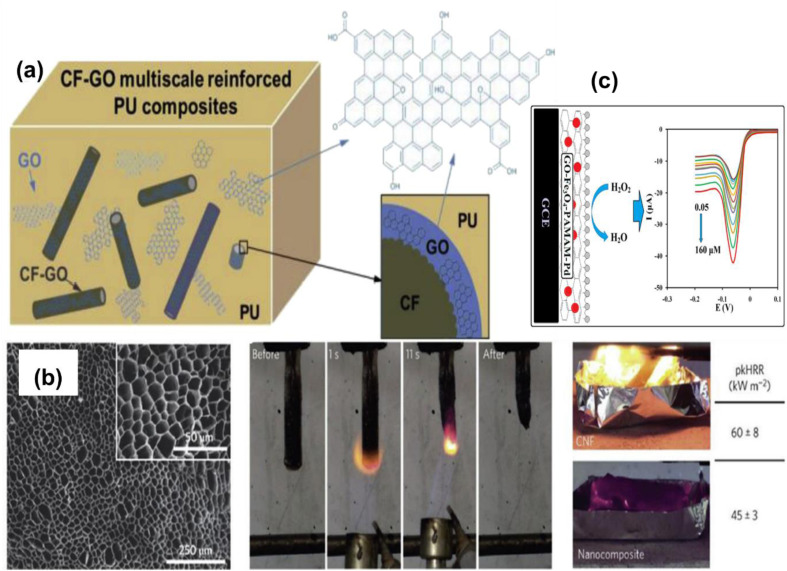
Properties of graphene oxide (GO) (a) mechanical properties; effect of carbon fibres reinforced with GO on polyurethane elastomer tensile strength (b) thermal properties; (i) SEM image of cellulose nanofibres, GO, sepiolite clay nanorods, and boric acid nanocomposite foam (BA); (ii) vertical burn test of a nanocomposite foam after application of a methane flame; (iii) CNF and CNF/GO/BA/SEP nanocomposite foams during the cone calorimetry test (c) magnetic properties; hydrogen peroxide selective detection by Pd NPs decorated magnetic GO. This figure has been reproduced from ref. 320, 333, 336 and 338 with permission from Elsevier, copyright 2025.

The mechanical characteristics of a pure single sheet of graphene, including 42 N m of break strength, and 1.0 TPa Young's modulus, with an intrinsic value of 130.5 GPa tensile strength.^142,314^ These attributes are due to the combination of GO and rGO because of surface groups and defects. GO itself and its derivatives are excellent fillers for polymer nanocomposites, in which a polyvinyl alcohol film with a 20% GO filler content has 59.6 MPa of tensile strength which is attributed to the GO filler strength and the matrix/filler interface due to the hydrogen bonding of OH group of polyvinyl and oxygen of GO.^315–319^ Jiang *et al.* described a blend of polyurethane containing GO and GO-reinforced carbon fibres, which increased the elastomer's tensile strength by 16.4% as shown in Fig. 14(a).^320,321^ The electrically conductive material graphene can improve the polymer conductivity at low filler concentrations. However, throughout the production process, the sp^2^ bonding orbitals are disrupted, making the material electrically resistive.^322,323^ To produce rGO, which retains residual sp^3^ linking carbon to oxygen but can improve electrical conductivity, researchers have experimented with reducing techniques.^324,325^ RGO is a substance that can potentially be used as a conductive filler in polymer matrices due to its improved properties.^326–328^ Graphene possesses a higher thermal conductivity in-plane, but its low thermal conductivity makes it a poor choice for most applications.^329,330^ However, the graphene content needs to be reduced before it can be incorporated into polymers. Generating rGO coatings can significantly boost in-plane thermal conductivity, which is useful in particular circumstances.^331,332^ Furthermore, GO improves the flame-retardant characteristics of polymer nanocomposites as depicted in Fig. 14(b). SEM images of the nano-composite foam exhibit very aligned and arranged pores in tubular form. When these were exposed to a vertical flame test, the self-propagation of the spark stopped in the foam, resulting in a 25% lower peak heat release rate (pkHRR) compared with the bare CNF foam.^333–335^ Superparamagnetic magnetic nanoparticles on graphene nanostructures are utilized in drug delivery, hyperthermia, and biosensing, and hydrogen peroxide detection is achieved through functionalization with amine groups at terminal positions in poly dendrimers and Palladium nanoparticles for the determination of selectivity of hydrogen peroxide as shown in Fig. 14(c).^336,337^

#### 4.3.1 Functional groups

Graphene oxide (GO) has a wide range of oxygen-containing functional groups, most notably hydroxyl, carboxyl, and epoxy moieties, which facilitate electrostatic attraction, hydrogen bonding, and coordination interactions. Because of these features, GO may form stable complexes with a wide range of metal ions and organic pollutants, making it an adaptable adsorbent for use in environmental applications.^339^ Graphite is often oxidised with strong oxidising chemicals such as KMnO_4_, HNO_3_, and H_2_SO_4_ to produce GO. Following oxidation, the material is exfoliated in water or other suitable organic solvents to form GO nanosheets.^340,341^ By adding reactive oxygenated functional groups over the surface, the oxidative procedure significantly enhances the physicochemical features of GO, including mechanical strength, electrical conductivity, chemical reactivity, and optical, thermal, and electrochemical behaviour. These surface functional groups operate as chemically active spots that may be changed or functionalized, either covalently or non-covalently, to tailor GO's surface chemistry to specific applications.^309^ Non-covalent interactions can be used to functionalize GO with organic moieties such as amine-based ligands or α,β-unsaturated carbonyl compounds, improving dispersion stability and structural integrity.^342^ Furthermore, heteroatom doping (such as N, S, and P) may be utilised to chemically change the surface of GO, creating novel functional sites such as C–S, C–P, and C–N groups. These doped variants make GO more helpful in a range of domains, including energy storage and catalysis, while also enhancing its adsorption properties. Notably, using codoping methods, phosphorus-doped graphene oxide (P@GO) has been developed as a promising supercapacitor electrode and efficient counter electrode material for solar cells.^343,344^ These alterations improve GO's surface reactivity and adsorptive affinity through electrostatic, hydrophobic, and π–π stacking interactions, making it more effective in removing new pollutants like micro- and nanoplastics.

### 4.4 Synthesis of graphene oxide

**Fig. 15:**
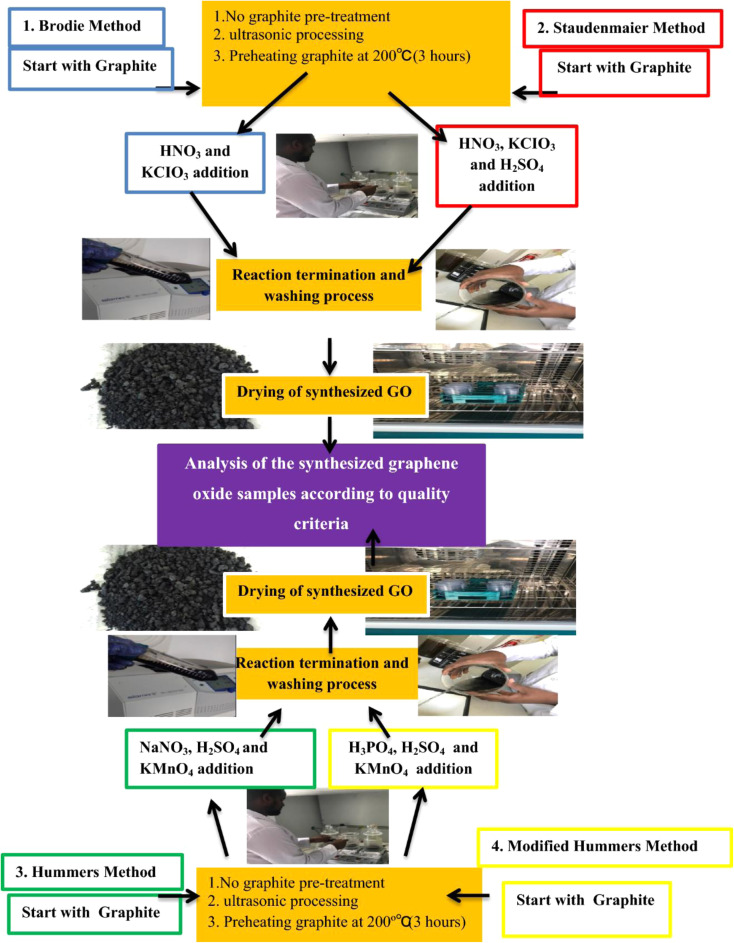
Synthesis of graphene by various methods, Preprint with permission. This figure has been reproduced from the ref. 346, with permission from Elsevier, copyright 2025.

We reviewed the processes developed by Staudenmaier, Brodie, and Hummer, and modified Hummers' method for the chemical oxidation of GO as depicted in Fig. 15.^345^ The impacts of several synthesis techniques, including the Brodie, Staudenmaier, Hummers, and Modified Hummers procedures, on graphite are discussed herein. Three graphite pretreatments were used: ultrasonication for five minutes to prevent structural flaws, and preheating for three hours at 200 °C to protect the bonding structure. The main purpose was to evaluate how numerous pre-treatments affect the synthesis techniques and quality standards for graphene oxide.^346^

#### 4.4.1 Brodie's method

In 1859, Brodie published the first method for creating graphene oxide (GO) by employing the chlorate pathway, using potassium chlorate as an oxidising agent. In his study, graphite was treated with strong oxidising agents such as potassium chlorate and fuming HNO_3_ at a temperature of 60 °C for 4 days to prepare GO and comprehend its structure.^347^ Brodie's method produces nanosheets that are rigid, having perfect microstructures.^348^ Korucu reported the formation of small quantities of oxidant by dumping of fuming nitric acid onto a combination of graphite and sodium/potassium chlorate.^346^ Feicht *et al.* prepared GO with a highly intact graphene lattice by successive oxidation of graphite, by dropping fuming HNO_3_ onto the blend of graphite and potassium or sodium chlorate. The carbon structure is maintained by varying the temperature of the reaction, leading to the production of graphene (oxo-G) functionalized with an oxo group, called a low-definition GO. Reductive defunctionalization can be used to transform this type of low-defect back into graphene.^349^ Talyzin *et al.* carried out graphite oxide synthesis employing the Brodie oxidation (BGO) method with one step, which produced GO flakes with a comparatively greater quantity of OH functional groups, whereas these groups are regularly distributed on the planar surface.^350^ Graphene oxides produced through the Brodie method result in exfoliation at 50–100 °C, higher temperatures and phase transitions among solvate phases of one and two layers. Among the two solvate phases, reversible phase transitions were observed by varying the temperature.^287,350,351^

**Fig. 16:**
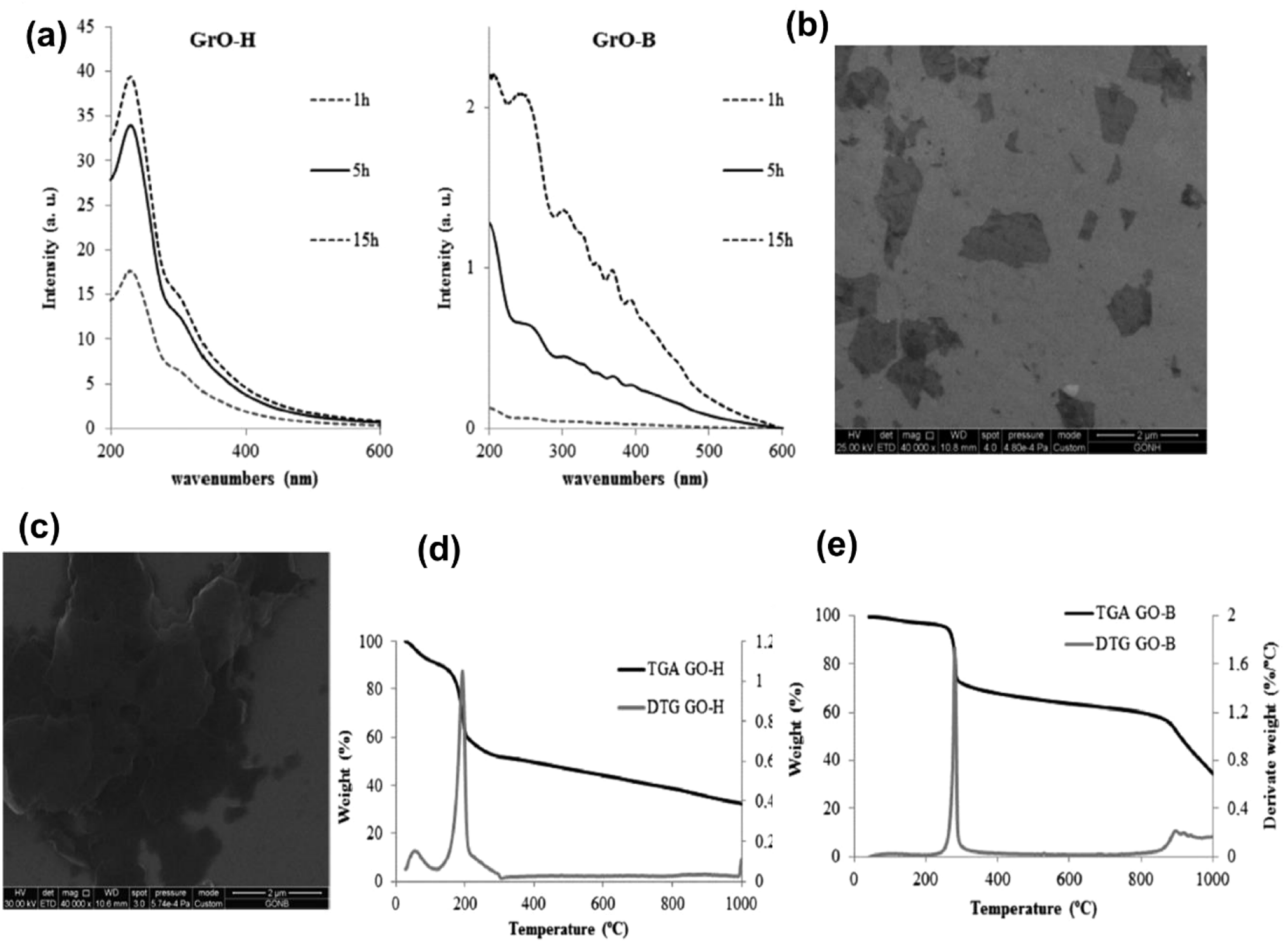
(a) UV-vis spectra of GO obtained through the Hummers' method and GO obtained through the Brodie method at different sonication times (b) SEM image of GO-H-5 h (shown at top right) and (c) SEM image of GO-B-5 h (bottom left) (d) TGA/DTG analysis of GO-H and (e) TGA/DTG analysis of GO-B, preprint with permission. This figure has been reproduced from the ref. 352, with permission from Elsevier, copyright 2025.

A smaller amount of oxygens are introduced in Brodie's method as compared to the Hummers' method, but Brodie's method favours conjugated epoxy and hydroxyl groups. These stable groups prevent the C sp^2^ structure's full recovery within the carbon lattice, while the Hummers oxidation method achieves a greater revival of the 2D structure of pure graphite. Exfoliated GOs obtained *via* the Hummers' method (GO-H) exhibit the characteristic peaks for GO in UV-vis adsorption spectra at 230 nm and 300 nm, which are due to the transitions from p–p orbitals of aromatic carbon to carbon and carbon to oxygen bonds, respectively. In contrast, GOs obtained through the Brodie method (GO-B) exhibit multi-peak formation over 300 nm, as illustrated in Fig. 16(a). The SEM and TEM figures reveal a greater quantity of monolayers obtained from GO-H shown in Fig. 16(b) and (c) The TGA/DTG curves of GO-H in Fig. 16(d) show that it starts losing weight at temperature below 150 °C and the maximum loss in weight occurs at temperature of 200 °C (40% weight loss). Whereas loss in weight reaches 54% at 800 °C temperature. Whereas no loss in weight occurs in GO-B until 200 °C temperature, while maximum loss in weight occurs at 250 °C (27% weight loss). Weight loss occurs up to the temperature of 900 °C, and afterwards a second maximum loss of weight (about 20%) up to a temperature of 900 °C. Subsequently, there is a secondary peak in weight loss (approximately 20%) observed between 900 °C and 1000 °C, as illustrated in Fig. 16(e). This implies that GO-B exhibits greater stability, indicating a lower quantity of oxygen-containing functional groups.^352–354^

Brodie oxidation improves hydrogen bonding and electrostatic interactions with negatively charged microplastics by introducing epoxy and hydroxyl functional groups. GO-B's selective adsorption performance and thermal stability are attributed to its reduced oxygen concentration and more organised structure. On the other hand, GO-H's enhanced surface area and functional group availability as a result of its greater oxygen content and defect density improve its overall adsorption capability. As a result, in water treatment applications, the synthesis technique has a direct influence on the structural and adsorption properties of GO.

#### 4.4.2 Staudenmaier method

Today's techniques for synthesising graphene oxide (GO) are improvements above those used by Brodie (1859), and Staudenmaier modified Brodie's method approximately 40 years later (in 1898) by altering the method of adding chlorate and sulfuric acid to the mixture to synthesise graphene oxide.^355^ Potassium perchlorate (KClO_4_) is the oxidising agent used in both procedures.^356^ The Staudenmaier technique synthesises graphene oxide from graphite by adding graphite, fuming HNO_3_, and H_2_SO_4_ to a glass reactor, followed by gradually adding KCIO_3_.^346^ This technique relies on the use of strong acids and oxidising agents for the oxidation of graphite. The process employed, the conditions of the reaction, and the characteristics of the graphite all affect the degree of oxidation.^357^ However, the methods utilised to generate graphene oxide (GO) require the use of harmful chemicals, and during this process, toxic gas is produced.^358^ Sheshmani & Fashapoyeh employed the modified Staudenmaier process for the preparation of GO using concentrated HNO_3_/H_2_SO_4_ in a 1 : 3 volume ratio, resulting in an improved degree of exfoliation.^359^ Sali *et al.* prepared GO by employing the Staudenmaier method, which produced a high content of highly polar carbonyl groups, resulting in an increase in the membrane's permeability and hydrophilicity in comparison with GO prepared by the Hummers and Tour methods. The hydrophilicity, adsorption affinity, and electrostatic interactions of GO with pollutants such as microplastics are directly caused by oxygen-containing functional groups (such as hydroxyl, carboxyl, and carbonyl) tailored by the oxidation process, which also influences the extent of exfoliation. For example, compared to hummer-derived GO, Staudenmaier-synthesized GO often has a larger density of polar carbonyl groups, which improves water permeability and selective adsorption capabilities.^360^

#### 4.4.3 Hummer's method

The Hummers' method is a conventional and effective procedure used for the synthesis of GO. This process was established by W. S. Hummers and R. E. Offeman.^361^ Hummers and Offeman enhanced their procedures by substituting excess potassium permanganate (KMnO_4_) for KClO_4_, sulfuric acid, and a small quantity of sodium nitrate. The time for the reaction ranged between 8 to 12 h.^362^ This approach is very safe because it avoids the production of explosive ClO_2._ Moreover, this method produces sheets of GO with large sizes, and it is more efficient at improving the mechanical characteristics of synthesised polysulfone (PSf) membranes. The high oxygen content and surface area of GO generated with this technology improve its interaction and dispersion in aquatic environments, hence enhancing adsorption and membrane-based water filtration efficiency.^360^

**Fig. 17:**
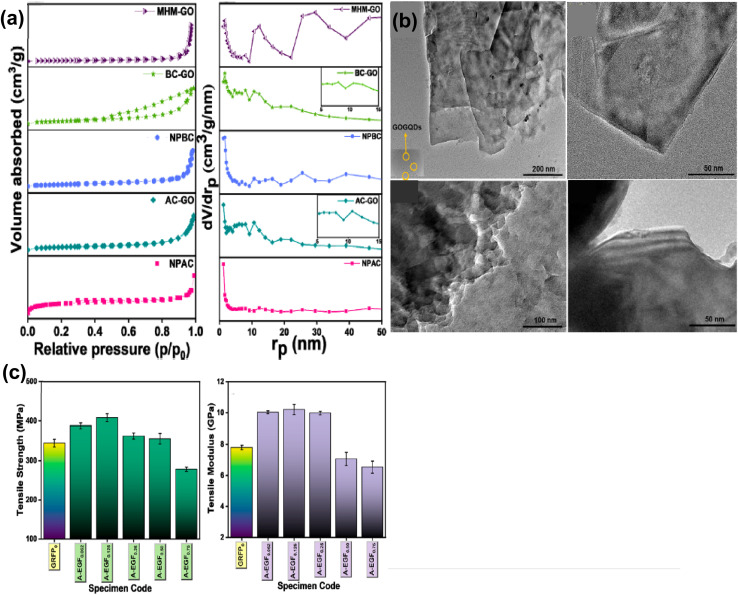
(a) BET nitrogen adsorption/desorption isotherms and BJH plots of GO from different coals and graphite (b) TEM images of AC-GO and BC-GO (c) mechanical properties; tensile strength and tensile modulus of AC-GO-based GFRP nanocomposites. This figure has been reproduced from the ref. 363, with permission from Elsevier, copyright 2025.

Grag *et al.* used the Hummers process for the one-pot preparation of graphene oxide employing HNO_3_. Fig. 17(a) shows the BET nitrogen adsorption–desorption isotherms and BJH plots for the GO acquired from multiple coals; the mesoporous nature of the samples and precursors was confirmed through BJH plots. The H1 hysteresis loop obtained in the case of BC-GO confirms the mesoporous nature of BC-GO. The TEM images show a multilayer structure for graphene oxide from AC-GO (semi-anthracite coal) and BC-GO (bituminous coal) samples, Fig. 17(b). The maximum level of mechanical properties was obtained when loading of GFRP nanocomposites at 0.125 phr with AC-GO (referred to as A-EGF_0.125_) was carried out, compared to the GFRP composite with no loading (GFRP_0_). The tensile strength was increased by 18.3%, while tensile modulus, flexure strength, and flexure modulus were enhanced by 30.9%, 22.7%, and 25.1%, respectively, as shown in Fig. 17(c).^363^ Venkatesan *et al.* used a typical procedure for making graphene from sub-bituminous coal.^364^ Das *et al.* obtained graphene oxide from demineralised coal by combining concentrated H_2_SO_4_ and NaNO_2_, and sonicating for a full day, and then HNO_3_ was added. Following treatment with NH_4_OH and ethanol, the remaining portion was dehydrated in a vacuum oven. The powdered form, which was obtained through grinding and scraping the flask's bottom phase.^365^ This study presents a different approach for synthesising graphene and GO from coal resources, offering the potential for energy storage and ecosystem preservation.^366^ Unfortunately, the Hummers' method is not environmentally friendly because of the evolution of NO_*x*_ during the reaction. Zhou *et al.* employed an eco-friendly and cost-effective Hummer's method for synthesising graphene oxide (GO), without H_2_O_2,_ by regulating the temperature and time of reaction and the amount of H_2_SO_4_. GO possesses a higher degree of oxidation, and it is rich in oxygen-containing groups in comparison to traditional methods.^367^ The importance of the synthesis process to water treatment operations is underscored by the fact that such oxygen-rich GO not only promotes increased adsorption of pollutants such as dyes and metal ions, but also improves compatibility and performance in membrane matrices.

#### 4.4.4 Modified Hummers' method

Numerous adjustments to Hummers' technique have been made to enhance yield and GO characteristics while minimising or eliminating drawbacks.^368^ To reduce the generation of hazardous gases, some investigations have employed a 9 : 1 acid mixture without NaNO_3_. According to another study, a 9 : 1 combination of KMnO_4_, H_2_SO_4,_ and H_3_PO_4_, and no sodium nitrate increased the GO yield and oxidation. It was discovered that GO made with K_2_Cr_2_O_7_ and a 2-hour reaction time had the lowest oxygen content.^358,369^ Chiang *et al.* predicted a novel technique for the production of graphene oxide from carbonised cellulose employing the modified Hummers' method.^370^ Chandio *et al.* synthesised GO by oxidising the graphite with potassium permanganate oxidising agent through modified Hummers' method after ozone treatment, which produced multilayer graphene oxide with variable thickness.^371^ The enhanced oxidation and multilayer structure improve GO adsorption effectiveness by increasing the density of oxygenated groups, improving interaction with ionic pollutants, and allowing for robust anchoring in membrane composites.

Guerrero-Contreras & Caballero-Briones investigated graphite oxidation to produce graphene oxide (GO) with different compositions of oxygen and ratios of oxygen to carbon. They employed different iterations of the Hummers' approach, altering the reactant ratio, reaction temperature, and reaction duration.^372^ These parameters have a significant impact on GO defect density and interlayer spacing, which are critical for modifying permeability, surface area, and pollutant-binding efficacy in water treatment membranes. Sierra *et al.* employed a modified Hummers' method using three cokes of petrochemical and carbon chemical sources to produce graphene oxide having crystalline structures of dissimilar sizes as raw materials.^366^ Purwandari *et al.* used Sawahlunto-Sijunjung coal and produced graphene using the modified Hummers Method, which is suggested to be an inexpensive and plentiful source of graphite.^373^ When used in membrane applications, the structural diversity of the resulting GO aids in the selective removal of contaminants while also providing differential water permeability. Graphene oxide synthesis methods were assessed, and minimum anticipated faults and low ratios of oxidation and structural defects were discovered. The most popular synthetic methodology, modified Hummers' method, has more structural flaws and oxygenated groups. The updated Hummers process better satisfies graphene oxide quality criteria and is economical and environmentally benign.^346^

Graphene oxide (GO) may be produced *via* a variety of oxidation processes, each of which has an effect on the material's oxygen concentration, adsorption capacity, and structural integrity. Strong oxidants are utilised in traditional techniques, like as Brodie's and Staudenmaier's, which produce GO with high thermal stability and well-preserved microstructures but take time and emit toxic gases. The later-developed Hummers process is more successful and popular due to higher oxidation levels and a quicker reaction time, but its NOx emissions continue to create environmental issues. The modified Hummers approach, on the other hand, employs safer acid combinations and avoids hazardous chemicals such as sodium nitrate, resulting in improved oxidation, surface functionalization, and increased GO output. According to the literature, the modified Hummers technique is most suited for producing GO as an effective adsorbent in micro/nanoplastics remediation because it offers the best mix of performance, scalability, and environmental safety.

## 5. Mechanism of adsorption of M/NPs with graphene oxide

**Fig. 18:**
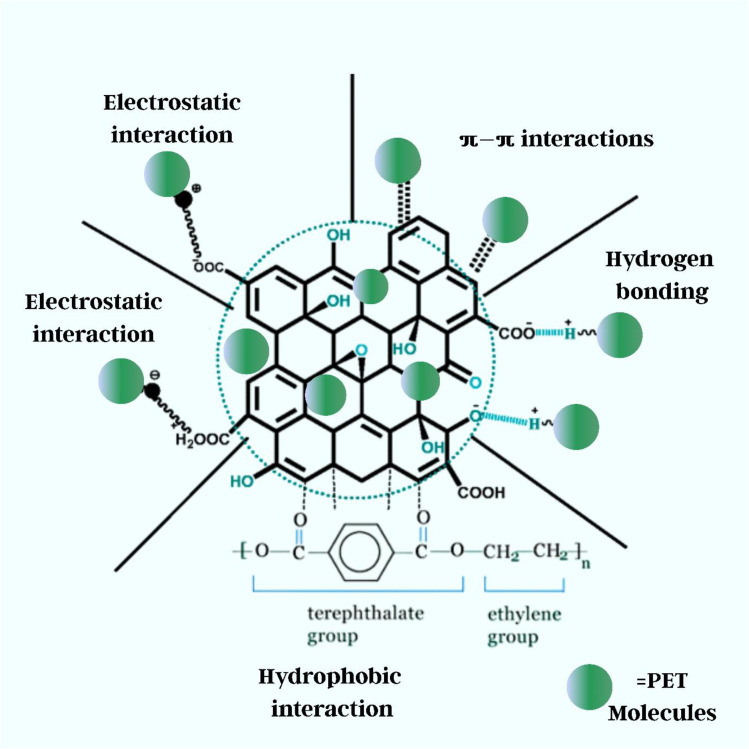
Mechanism of M/NPs adsorption on GO.

There are essentially five ways for graphene oxide to interact when employed as an adsorbent to remove micro and nanoplastic pollution: (i) pi–pi stacking interactions, (ii) van der Waals interactions, (iii) hydrophobic interactions, (iv) hydrogen bonding (v) electrostatic interactions, as shown in Fig. 18. When in contact with water, stable colloidal dispersions can form and interact with water through a hydration mechanism. Graphene oxide, which is derived from graphene, is hydrophilic and contains carboxyl, hydroxyl, epoxy, and functional groups, including oxygen.^374^

First, pi–pi stacking interactions occur when aromatic domains form when π–electrons are delocalized in GO's graphene basal plane. Aromatic moieties of plastic polymers, such as polystyrene (PS) and polyethene terephthalate (PET), can interact with π–electrons. The π–π stacking interaction between GO's aromatic rings and plastic polymers promotes significant affinity and stable adsorption of aromatic-rich microplastics onto the GO surface.^375–377^ Another type of van der Waals interaction occurs when weak and non-specific interactions occur between nonpolar polymer chain segments and hydrophobic portions of the GO sheets. van der Waals forces are enhanced by the size of the polymer molecules, their accessible surface area, and the physical closeness of the polymer surfaces to GO. These characteristics contribute to the physical trapping and adhesion of microplastic impurities on GO surfaces.^378,379^ GO is hydrophilic due to its oxygenated groups, although there are hydrophobic patches in the sp^2^ hybridised areas with graphitic carbon domains. Nonpolar microplastic pieces preferentially cling to hydrophobic GO patches, reducing their exposure to the aqueous environment.^380,381^

In hydrogen bonding, oxygen-containing functional groups in GO sheets include hydroxyl (–OH), epoxy (–O–), and carboxyl (–COOH), which can form hydrogen bonds with polar functional groups on micro/nanoplastics (*e.g.*, –OH, –NH_2_, –COOH). This promotes strong directed interactions and selective adsorption, especially when the surface of microplastics is functionalized or oxidised.^382^ In electrostatic interaction, the ionisation of its oxygenation groups gives GO a negative surface charge in aqueous environments. This allows GO to interact electrostatically with micro/nanoplastic regions that are negatively or positively charged. The stability of GO dispersion and adsorption efficiency are affected by these interactions, which change dynamically with environmental parameters such as pH and ionic strength.^76,383^ Finally, due to its hydrophilic nature and colloidal stability in water, GO may form homogenous dispersions that improve surface contact with pollutants *via* hydration and interfacial interactions. These combined methods highlight GO's broad-spectrum affinity, making it one of the most adaptable and effective materials for water micro/nanoplastic cleanup. In addition to these generic adsorption pathways, the adsorption of M/NPs onto GO is mainly governed by a balance between chemisorption and physical adsorption processes; the interaction strength depends strongly on both the plastic type and the surrounding aqueous conditions. Chemisorption mechanisms, such as surface complexation, hydrogen bonding, and covalent-like interactions, play a particularly important role in securing stable attachment of plastics onto GO. For example, polar polymers such as nylon (polyamide) and oxidised polyethene terephthalate (PET) interact strongly through hydrogen bonding between amide or ester groups and the hydroxyl or carboxyl functionalities of GO. In acidic media, protonated amine groups in nylon further interact electrostatically with deprotonated GO carboxylates, while in saline conditions, cation bridging may stabilise PET–GO complexes. These chemisorptive interactions are accompanied by physical adsorption pathways, including van der Waals forces, π–π stacking, hydrophobic association, and pore filling. In particular, aromatic polymers such as polystyrene (PS) and PET exhibit strong π–π stacking with the graphitic domains of GO, facilitating stable immobilisation of these polymers under neutral pH.^185^ Nonpolar plastics such as polyethene (PE) and polypropylene (PP), which lack aromatic or polar groups, depend mainly on hydrophobic interactions with the sp^2^ domains of GO. However, ageing and photooxidation can introduce polar moieties (–OH, –COOH) to PE and PP surfaces, enhancing their affinity *via* hydrogen bonding and electrostatic attraction. Halogenated polymers such as PVC, although less interactive, can adsorb through electrostatic interactions between the negatively charged oxygenated groups of GO and the partially polarised C–Cl bonds.^381^

Beyond polymer chemistry, the structural characteristics of GO significantly influence adsorption efficiency. The high surface area and heterogeneous pore structure of GO provide abundant binding sites where both electrostatic forces and hydrophobic attractions can occur simultaneously. Micropores enhance the overall adsorption capacity by increasing accessible surface area, while mesopores promote faster diffusion of polymer fragments, thereby accelerating adsorption kinetics. This dual pore system ensures that both small nanoplastics and larger microplastic fragments are effectively captured. In water matrices, the polarity and heterogeneity of GO's surface are critical, as they facilitate ion–dipole interactions and electrostatic attraction of charged plastic surfaces. Importantly, while physical adsorption processes such as pore filling and van der Waals forces contribute to initial capture, they are relatively weak compared to chemisorption, which ensures more stable and selective removal of M/NPs under variable environmental conditions.^379^ The interplay of these mechanisms highlights that GO adsorption is not governed by a single process but rather by a spectrum of interactions modulated by polymer type, water chemistry (pH, ionic strength, and natural organic matter), and the textural properties of the adsorbent. Having described the adsorption mechanisms in detail, the following section reviews experimental demonstrations of GO-based materials in M/NP remediation.

## 6. Graphene oxide as adsorbent for M/NPs remediation

Graphene oxide (GO)-based materials have shown considerable potential in the remediation of micro/nanoplastics. For example, Uogintė *et al.* reported that GO-metal oxide nanocomposites degraded polyethene microplastics under UV light by up to 50.46%, as validated by FTIR and following pseudo-first-order kinetics.^384^ A multifunctional SA/GO/CS membrane cleaned oils, dyes, and nanoplastics with over 99% efficiency and great reusability *via* adsorption, sieving, and charge interactions.^385^ Molecular docking revealed strong GO binding to BPA and PET through hydrogen bonding and π–π stacking.^386^ Yan *et al.* developed a 99.9%-efficient reduced GO (S-rGO) membrane for 200 nm MPs, displaying high water flow and mechanical stability.^380^ Ko *et al.* developed a reusable GO/CS/Genipin sponge capable of removing up to 73% of nanoplastics *via* hydrophobic and electrostatic interactions.^387^ Vijayshanthy *et al.*'s PVA/GO membrane eliminated 84% of MP from WWTPs *via* IoT-based monitoring while also reducing turbidity, BOD, and other pollutants, as shown in Table 3.^388^

**Table 3:** Overview of graphene oxide-based adsorbents for micro/nanoplastics (M/NPs) remediation

Type of plastic	GO form used	pH range	Removal efficiency (%)	References
Polyethylene	GO–Cu_2_O	3–5	48.06%	384
Polyethylene	GO–MnO_2_	3–5	39.54%	384
Polyethylene	GO–TiO_2_	3–5	50.46%	384
Nanoplastics	Sodium alginate/GO/chitosan	Not specified	97.10%	385
Microplastics (200 nm)	Reduced graphene oxide membrane	Not specified	99.9%	380
26 nm nanoplastics	GO/chitosan/genipin sponge	5.5–7	73.0%	387
Polystyrene MPs	GO/chitosan/genipin sponge	5.5–7	41.5%	387
Mixed microplastics	PVA/graphene oxide membrane	6.90–7.95	84%	388

**Fig. 19:**
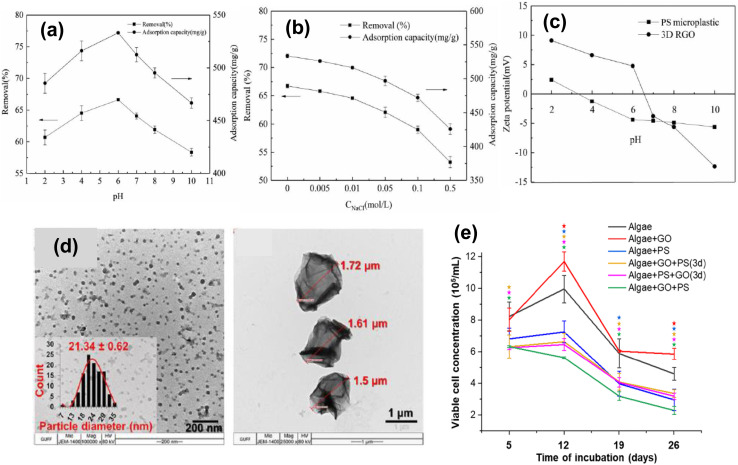
(a) Effect of different initial concentrations on the adsorption of PS microplastics on 3D RGO. (b) Zeta potentials of 3D RGO and PS microplastics at different pH values. (c) Effect of different adsorption times on the adsorption of PS microplastics on 3D RGO (d) TEM image of NPs removal by GO (e) effect of GO and 20 nm PS NPs on *Picochlorum* sp. microalgae on a viable cell concentration as a function of time, this figure has been reproduced from ref. 390 with permission from Elsevier, copyright 2025.

According to the study, the 3D RGO surface morphology includes more surface area, multiple pore structures, a high degree of peeling, and a fluffy look. Microplastic adsorption increases as pH rises, and its removal efficiency and adsorption capacity also increase, highest at pH 6. The isoelectric points of the PS microplastics were acidic. In this study, PS microplastics and 3D reactive glass (RGO) zeta potential are investigated at various pH levels, as shown in Fig. 19(b). These findings demonstrated the positive charge of 3D RGO, which limited its adsorption capacity and removal efficiency. However, the modest negative charge of the polystyrene microplastics enhanced their ability to adsorb. A higher negative charge at pH 6 led to enhanced adsorption capacity and removal efficiency. However, negatively charged PS microplastics lost their adsorption capability and effectiveness at pH 7.^389^ At various starting concentrations, the process of adsorption of 3D RGO on PS microplastics is shown in Fig. 19(a). A 600 mg L^−1^ concentration was the ideal concentration. Following the adsorption of PS microplastics, SEM and XRD studies examined the surface conformation of 3D reactive glass (RGO). The strength of the π–π interlinkage between the aromatic ring of the polystyrene microplastics and the carbon ring of 3D RGO was found to be enhanced due to adsorption. In the initial 30 minutes, the microplastics' elimination efficiency surged from 28.71% to 54.35%, and between 30 to 120 minutes, it reached 66.10%. The removal efficacy and adsorption capacity of 3D RGO exhibited stability beyond the 120-minute mark, indicating equilibrium, as depicted in Fig. 19(c). The movement method of PS microplastics onto the surfaces of 3D RGO was further investigated using the intraparticle diffusion model. Particle internal diffusion and membrane dispersion are the two categories of adsorption processes. With increasing temperatures, the removal effectiveness of microplastics has risen from 66.83% to 72.63%. Simultaneously, the adsorption capacity of 3D RGO has increased from 534.60 mg g^−1^ to 580.98 mg g^−1^. These results indicate that elevated temperatures enhance the adsorption efficiency of 3D RGO toward microplastics.^53^ Yesilay *et al.* conducted a study exploring the utilisation of graphene oxide (GO) as a coating material. They evaluated its effectiveness in mitigating the toxicity of polystyrene nanoparticles (PS NPs) on microalgae that had been treated with GO. The TEM image is shown in Fig. 19(d) zeta potential was found to be −35.7 mV, and PS NPs had a diameter of 20 nm. However, TEM imaging of GO displayed sheets that are 1.5 μm in diameter. Results show that the toxicity of PS NPs was reduced by the treatment of microalgae with GO. The highest growth inhibition rate (IR%) values were observed at 50% for the algae + GO + PS group on a particular day and 26% for the algae + PS + GO (3d) group, shown in Fig. 19(e).^390^ These findings confirm GO's versatility and reusability, which serve as the foundation for evaluating practical challenges and future directions.

## 7. Current challenges and future perspective

Graphene oxide (GO) shows tremendous potential as an adsorbent for the remediation of micro- and nanoplastics due to its enormous surface area, abundance of functional groups, and strong pollutant attraction. Nonetheless, several concerns remain unsolved. While GO can be synthesised on a large scale at relatively low cost, uncertainties remain regarding its long-term stability, regeneration, and reusability in practical water treatment systems. Furthermore, major concerns remain concerning GO's potential toxicity and environmental impact after usage. Future studies should focus on optimising GO synthesis strategies to boost yield and functionalization while lowering environmental impact. Furthermore, GO's performance and recovery may be enhanced by combining it with other technologies such as membrane filtration, photocatalysis, or magnetic separation. The development of GO-based nanocomposites containing metal oxides, carbon-based materials, or biopolymers has a high potential for synergistic effects, which might improve M/NP adsorption capacity and photocatalytic degradation. Bridging the gap between effective laboratory-scale operations and realistic field-scale deployment requires interdisciplinary initiatives focused on eco-friendly synthesis, life-cycle evaluation, and pilot-scale testing. With these discoveries, GO and its derivatives may give realistic and scalable solutions to minimise microplastic pollution in aquatic ecosystems.

### 7.1 Selectivity and limitations in complex matrices

In real-world systems such as wastewater treatment plants (WWTPs), the adsorption performance of GO towards M/NPs is considerably more complex than under controlled laboratory conditions. While laboratory studies consistently highlight strong π–π stacking, electrostatic, and hydrophobic interactions driving M/NP adsorption onto GO, the presence of natural organic matter (NOM), salts, and diverse co-contaminants in wastewater significantly modulates these interactions. NOM, particularly humic and fulvic acids, competes with plastic surfaces for GO's oxygenated functional groups, while also imparting steric hindrance that reduces effective surface contact. High ionic strength environments, common in municipal and industrial effluents, compress the electrical double layer around GO, diminishing long-range electrostatic attraction with charged plastics such as polyamide or oxidised PET.^388^ Multivalent cations (Ca^2+^, Mg^2+^, Fe^3+^), abundant in real wastewater, can further shield GO's surface charges or induce cation bridging, leading to aggregation of GO-M/NP complexes that alter adsorption pathways. This aligns with findings from WWTP surveys across Iran, Australia, and Europe, where polyethene, polypropylene, and polyester fibres were detected even after secondary treatment, indicating that hydrophobic plastics in particular face strong competition from oils, surfactants, and colloidal organics for GO's hydrophobic binding domains. Moreover, particle size introduces another layer of selectivity: nanoplastics (<100 nm) can diffuse into GO mesopores, while larger microplastic fragments (>500 μm) rely primarily on surface adhesion, a process strongly influenced by wastewater turbulence, suspended solids, and biofilm formation.^391–394^ Evidence from WWTP studies further demonstrates that smaller MPs are enriched during treatment due to mechanical fragmentation, thus increasing the fraction of particles most likely to interact with GO but also exacerbating analytical challenges in separation and recovery.^388^

Compared with conventional adsorbents such as activated carbon and biochar, GO offers unique advantages in terms of surface functionality and tunable interactions. Activated carbon mainly relies on physical adsorption through van der Waals forces and pore filling, processes that are strongly dependent on surface area and pore distribution but often lack selectivity towards specific polymer types. In contrast, GO provides a chemically heterogeneous surface with abundant carboxyl, hydroxyl, and epoxy groups capable of engaging in hydrogen bonding, electrostatic interactions, and even covalent bonding under oxidative conditions. This enables stronger and more selective affinity for functionalized or aromatic plastics such as polystyrene and PET. However, while activated carbon and biochar display robust stability and reusability under complex effluent conditions, GO's performance is more sensitive to pH fluctuations, ionic strength, and NOM fouling, which can diminish its adsorption capacity.^395^ These comparative insights underscore that although GO exhibits superior mechanistic versatility and higher removal efficiencies under controlled conditions, its application to full-scale wastewater treatment requires composite designs or hybrid systems that combine the selectivity of GO with the stability of conventional adsorbents.

## 8. Conclusion

This review highlights the various sources, pathways of micro- and nanoparticles (M/NPs) in the environment, with a particular emphasis on wastewater and the toxicological effects of these particles. Furthermore, this study provides a detailed summary of the several approaches that the scientific community has recently employed to reduce MNP pollution in wastewater through bioremediation. The effects of plastic particles, especially microplastics, and remediation methods, including chemical, biological, and hybrid approaches, were thoroughly discussed. In this review, several MNP remediation techniques and technologies have been systematically described. We have discussed the adsorption method for our primary investigation by using graphene oxide as an adsorbent. To achieve optimal results, both graphene oxide and 3D reduced graphene oxide can be exploited. We deliberated on the mechanism of graphene oxide interacting with micro nanoplastic-polluted water, encompassing electrostatic forces, π–π stacking, hydrogen bonding, hydrophobic interactions, and van der Waals forces. Overall, GO-based strategies show strong potential for scalable and efficient microplastic remediation in future.

## Conflicts of interest

The authors declare no competing interests.

## Data Availability

No new experimental data, software, or code were generated or analysed in the preparation of this review. All relevant information, including data presented in figures and tables, has been obtained from previously published studies, which are cited throughout the article.
